# Endosymbionts Reduce Microbiome Diversity and Modify Host Metabolism and Fecundity in the Planthopper *Sogatella furcifera*

**DOI:** 10.1128/msystems.01516-21

**Published:** 2022-03-30

**Authors:** Tong-Pu Li, Chun-Ying Zhou, Meng-Ke Wang, Si-Si Zha, Jie Chen, Xiao-Li Bing, Ary A. Hoffmann, Xiao-Yue Hong

**Affiliations:** a Department of Entomology, Nanjing Agricultural University, Nanjing, China; b School of BioSciences, Bio21 Institute, The University of Melbourne, Victoria, Australia; University of Connecticut

**Keywords:** *Sogatella furcifera*, *Cardinium* and *Wolbachia*, microbiome, metabolite, correlation, tissue-specific, bacterial microbiota

## Abstract

Endosymbionts can strongly affect bacterial microbiota in pests. The white-backed planthopper *Sogatella furcifera*, a notorious pest in rice, is usually co-infected with *Cardinium* and *Wolbachia*, but the effects of these endosymbionts together or individually on the host microbiome and fecundity are unclear. Here, we established three *S. furcifera* lines (*Cardinium* and *Wolbachia* double-infected, *Cardinium* single-infected, and both-uninfected lines) backcrossed to a common nuclear background and found that single and double infections reduced bacterial diversity and changed bacterial community structure across nymph and adult stages and across adult tissues. The endosymbionts differed in densities between adults and nymphs as well as across adult tissues, with the distribution of *Cardinium* affected by *Wolbachia*. Both the single infection and particularly the double infection reduced host fecundity. Lines also differed in levels of metabolites, some of which may influence fecundity (e.g., arginine biosynthesis and nicotinamide metabolism). *Cardinium* in the single-infected line upregulated metabolic levels, while *Wolbachia* in the double-infected line appeared to mainly downregulate them. Association analysis pointed to possible connections between various bacteria and differential metabolites. These results reveal that *Cardinium* by itself and in combination with *Wolbachia* affect bacterial microbiota and levels of metabolites, with likely effects on host fecundity. Many of the effects of these metabolically limited endosymbionts that are dependent on the hosts may be exerted through manipulation of the microbiome.

**IMPORTANCE** Endosymbionts can profoundly affect the nutrition, immunity, development, and reproduction of insect hosts, but the effects of multiple endosymbiont infections on microbiota and the interaction of these effects with insect host fitness are not well known. By establishing *S. furcifera* lines with different endosymbiont infection status, we found that *Cardinium* and the combined *Cardinium* + *Wolbachia* infections differentially reduced bacterial diversity as well as changing bacterial community structure and affecting metabolism, which may connect to negative fitness effects of the endosymbionts on their host. These results established the connections between reduced bacterial diversity, decreased fecundity and metabolic responses in *S. furcifera*.

## INTRODUCTION

Facultative mutualist microorganisms play an important role in many physiological processes of invertebrates, such as nutrition (1, 2), immunity (3, 4), development (5, 6) and reproduction (7, 8). The microbiota of invertebrates includes heritable intracellular symbiotic bacteria (i.e., endosymbionts) which live inside host cells, as well as other bacteria that live outside host cells or in the enteric cavity. Endosymbionts often influence the community of other bacteria in their hosts (9, 10). *Wolbachia*, the most widely distributed endosymbiont in arthropods, is known to have such effects in the small brown planthopper *Laodelphax striatellus* (11), fruitfly Drosophila melanogaster (12) and tick Ixodes pacificus (13). In addition to *Wolbachia*, the endosymbiont *Rickettsia* can change bacterial abundance in the flea *Ctenocephalides felis* (10), while the endosymbiont *Cardinium* can reduce bacterial diversity in the brown planthopper *Nilaparvata lugens* (14).

The fitness consequences of endosymbiont infections may be partly mediated by their impacts on the host microbiome. So far, it has been established that traits like reproduction can be influenced by endosymbionts in complex ways (15, 16). *Wolbachia* infections increase fecundity in *N. lugens* (17), *L. striatellus* (17), *Drosophila suzukii* (18) and the parasitic wasp *Trichogramma brassicae* (19), but they reduce fecundity in other species, including Aedes albopictus (20) and Drosophila simulans (21). Other endosymbionts like *Cardinium* can also reduce fecundity as documented in *N. lugens* (14). The extent to which these effects are mediated through changes in the microbiota are unclear but possible; bacterial community changes have been shown to reduce the fecundity of spider mites on multiple plants (22) and to also affect the fecundity of the fruit fly, *Bactrocera tryoni* (23).

In the field, hosts are often co-infected with multiple endosymbionts. For example, the parasitic wasp *Spalangia endius* (24), nematode *Pratylenchus penetrans* (25) and thrips *Pezothrips kellyanus* (26) are usually co-infected by *Cardinium* and *Wolbachia*, while the spider mite *Tetranychus truncatus* (27) is usually co-infected by *Wolbachia* and *Spiroplasma*. The impacts of co-infections by multiple endosymbionts on pest fecundity are complex (27, 28). For example, *Wolbachia* and *Spiroplasma* in the spider mite *T. truncatus* have different effects on host fecundity when they co-occur than when they are present as single infections (27). The effects of multiple endosymbionts on the host microbiome (and the interaction of these effects with host fitness) are not well known.

The white-backed planthopper, *Sogatella furcifera*, is usually co-infected with *Cardinium* and *Wolbachia*, both of which can induce cytoplasmic incompatibility (CI) (29, 30). This pest severely damages rice by laying eggs, sucking stalks, and transmitting plant viruses (31). In this study, we explored the effects of *Cardinium* and *Wolbachia* infections on the bacterial microbiota of *S. furcifera* at different developmental stages and in different adult tissues. The relative densities and distributions of *Cardinium*, *Wolbachia* and the total bacterial load were measured. We also determined the effects of endosymbionts on the host's metabolic levels, and we explored links between these effects and host fecundity.

## RESULTS

### *Cardinium* and the combined *Cardinium* + *Wolbachia* infections reduce bacterial diversity and change bacterial community structure.

**FIG 1:**
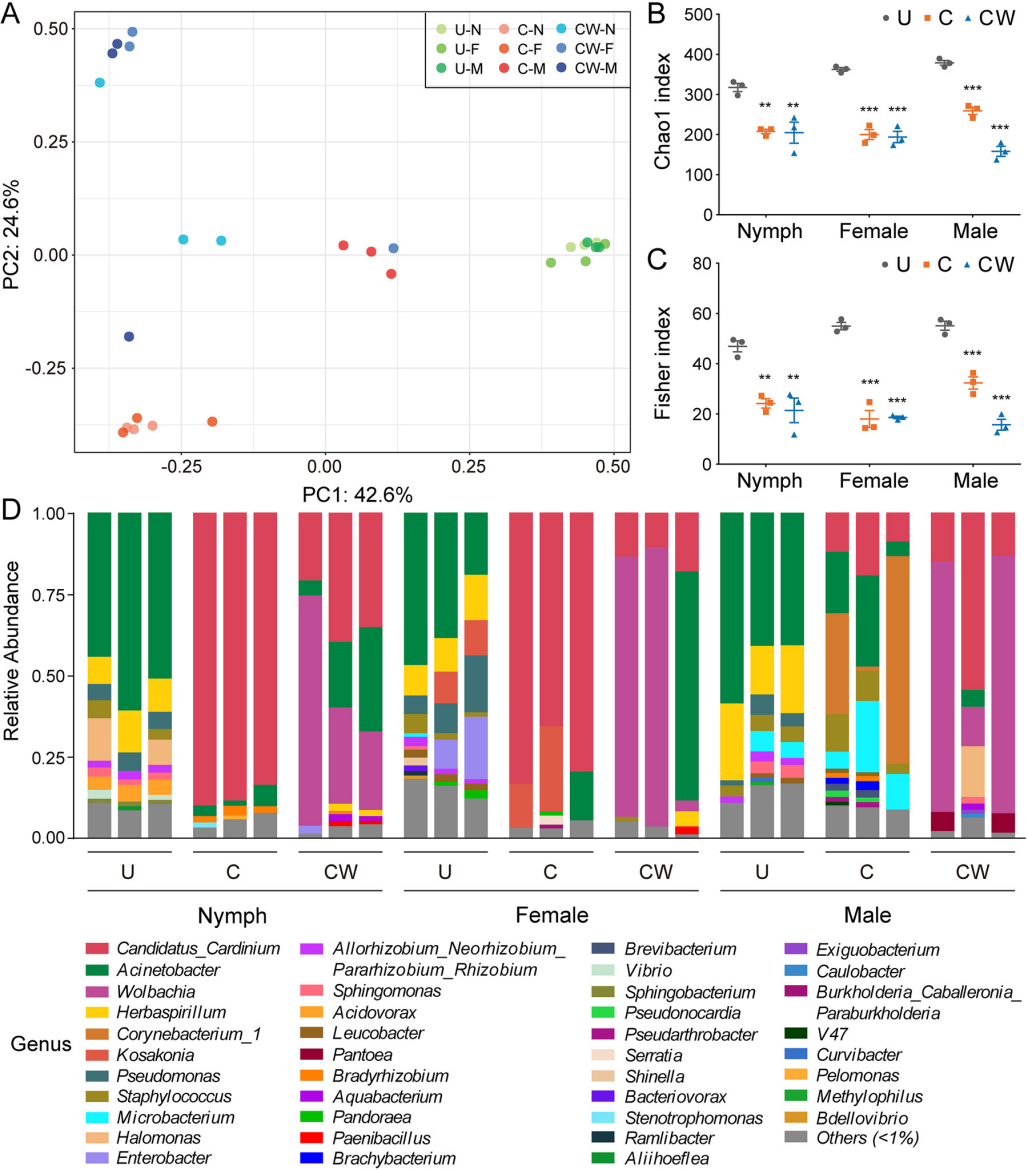
Bacterial diversity and relative abundance across different developmental stages in the three *S. furcifera* lines. (A) Principal coordinates analysis (PCoA) showing bacterial OTU-based clusters. Circles with different colors represent different samples, *n *= 3. (B and C) Scatterplots representing Chao1 (B) and Fisher (C) indexes of bacterial diversity. Asterisks indicate significant differences between lines, *n *= 3; *, *P* < 0.05; **, *P* < 0.01; ***, *P* < 0.001. (D) Relative abundance of bacteria showing bacterial community structure at genus level. Bacteria with a relative abundance of less than 1% were classified as Others. U, uninfected *S. furcifera* line; C, *Cardinium* single-infected *S. furcifera* line; CW, *Cardinium* and *Wolbachia* double-infected *S. furcifera* line; N, nymph; F, female; M, male.

The bacterial diversity of whole insect body in *S. furcifera* was affected by the presence of the endosymbionts and differed between developmental stages. There was clear separation of the samples of whole insect body at different developmental stages between the three lines (U, C, CW) in a PCoA plot (PERMANOVA: *F *= 8.92, R-squared = 0.799 and *P* < 0.001) (Fig. 1A). For each developmental stage, the Chao1 index (reflecting species richness of bacteria) of C or CW compared with U was significantly affected (one-way ANOVA: nymph, *P* < 0.001; female, *P* < 0.001; male, *P* < 0.001) (Fig. 1B). The Chao1 index of CW tended to be lower than C in males (one-way ANOVA followed by Tukey's pairwise comparisons, *P* < 0.001), but not in nymphs (*P* = 0.991) or females (*P* = 0.920). Results for the Fisher index (reflecting species richness and evenness of bacteria) between different lines matched those for the Chao1 index (Fig. 1C).

The adult tissues in the three lines were also significantly separated in the PCoA (PERMANOVA: *F *= 10.27, R-squared = 0.820 and *P* < 0.001) (Fig. S3A). For each tissue, the changes of Chao1 and Fisher indexes between the three lines were consistent (Fig. S3B-C). The average values of bacterial diversity in C and CW were lower than that of U, which was particularly marked in the testis (one-way ANOVA: Chao1, *P* < 0.001; Fisher, *P* < 0.05). Bacterial diversity in various tissues was not significantly different between C and CW. These results indicate that both single and double infections reduced bacterial diversity in each developmental stage, and that the double infection had a stronger effect than the single infection in males. This reduction was also reflected in adult tissues, especially in testis.

10.1128/msystems.01516-21.3 FIG S3 Bacterial diversity and relative abundance in the adult tissues of three *S. furcifera* lines. (A) Principal coordinates analysis (PCoA) showing bacterial OTU cluster. Circles with different colors represent different samples. *n *= 3. (B and C) Scatter plots representing Chao1 (B) index and Fisher (C) indexes of bacterial diversity. Asterisks indicating significant difference, *n *= 3; *, *P* < 0.05; **, *P* < 0.01; ***, *P* < 0.001. (D) Relative abundance of bacteria showing bacterial community structure at genus-level. Bacteria with a relative abundance of less than 1% were classified as Others. U, uninfected *S. furcifera* line; C, *Cardinium* single-infected *S. furcifera* line; CW, *Cardinium* and *Wolbachia* double-infected *S. furcifera* line; O, ovary; T, testis; G, gut. Download FIG S3, TIF file, 1.9 MB. Copyright © 2022 Li et al. 2022 Li et al. https://creativecommons.org/licenses/by/4.0/ This content is distributed under the terms of the Creative Commons Attribution 4.0 International license.

The bacterial community structure in the whole insect body of *S. furcifera* was significantly affected by endosymbionts. Eight phyla were identified in samples of different developmental stages in the three lines (Fig. S1A). The relative abundance of Proteobacteria was highest (60.51%), followed by Bacteroidetes (28.73%), Actinobacteria (7.76%), Firmicutes (2.7%), Patescibacteria (0.15%), Cyanobacteria (0.11%), Verrucomicrobia (0.04%), and Acidobacteria (<0.01%). Bacterial community structure differed in lines and among developmental stages (Fig. 1D and Fig. S2A). *Cardinium* and *Wolbachia* were dominant in C and CW, except for males in C. Microbiome analysis found that Acinetobacter was the major core member of the microbiome in U (Fig. S1B) and was also the major core member of the microbiome in addition to the endosymbionts in C and CW (Fig. S1C-D).

10.1128/msystems.01516-21.1 FIG S1 Microbiome profile in the three *S. furcifera* lines. (A) Pie chart showing the overall bacterial structure at phylum-level. (B-D) Heatmap depicting the core microbiome at genus level in U (B), C (C) and CW (D). U, uninfected *S. furcifera* line; C, *Cardinium* single-infected *S. furcifera* line; CW, *Cardinium* and *Wolbachia* double-infected *S. furcifera* line. Download FIG S1, TIF file, 1.4 MB. Copyright © 2022 Li et al. 2022 Li et al. https://creativecommons.org/licenses/by/4.0/ This content is distributed under the terms of the Creative Commons Attribution 4.0 International license.

10.1128/msystems.01516-21.2 FIG S2 Bacterial relative abundance depicting phylum-level bacterial community structure in three *S. furcifera* lines. (A) Different developmental stages. (B) Adult tissues. U, uninfected *S. furcifera* line; C, *Cardinium* single-infected *S. furcifera* line; CW, *Cardinium* and *Wolbachia* double-infected *S. furcifera* line. Download FIG S2, TIF file, 1.7 MB. Copyright © 2022 Li et al. 2022 Li et al. https://creativecommons.org/licenses/by/4.0/ This content is distributed under the terms of the Creative Commons Attribution 4.0 International license.

The differences in bacterial structure in the three lines were evident in adult tissues (Fig. S2B and S3D), although the bacterial structure differed in the tissues. The two endosymbionts comprised more than 75% of the relative abundance of bacteria in the CW gut. Overall, both single and double infections reduced the relative abundance of other bacteria and changed the bacterial community.

### *Cardinium* and the combined *Cardinium* + *Wolbachia* infections change the correlation between bacterial microbiota.

**FIG 2:**
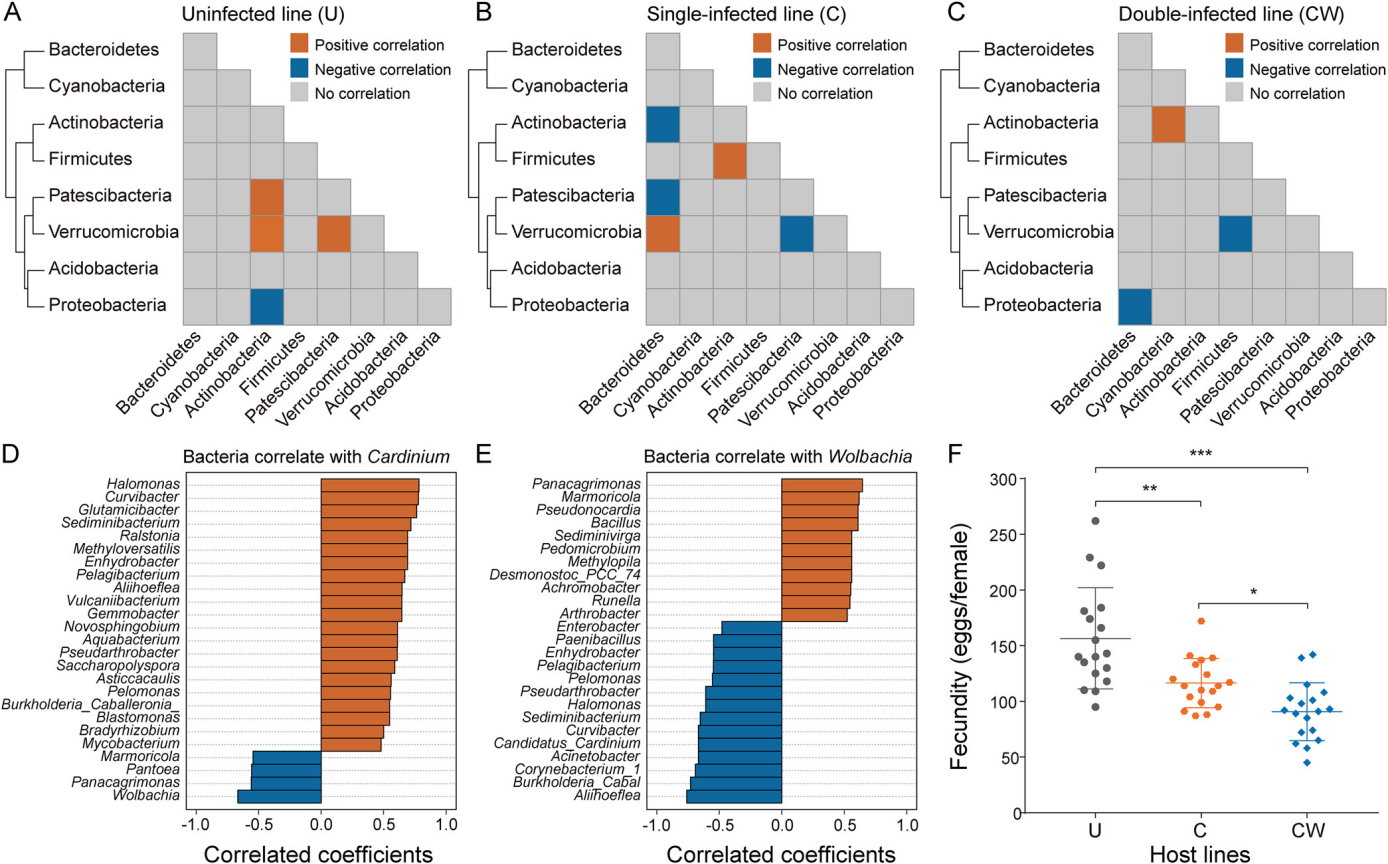
Correlation analysis of bacteria and host fecundity determination in the three *S. furcifera* lines. (A–C) Pairwise co-occurrence patterns of bacterial microbiota at phylum-level in U (A), C (B) and CW (C). Red, blue and gray indicate positive correlation, negative correlation and no correlation between the two bacterial phyla, respectively. (D and E) Genus-level correlation of *Cardinium* (D) and *Wolbachia* (E) with other bacteria in double-infected line (CW). (F) Fecundity effects of the three lines. Asterisks indicate significant difference the two compared group, *, *P* < 0.05; **, *P* < 0.01; ***, *P* < 0.001. Data were expressed as mean ± SD. U, uninfected *S. furcifera* line; C, *Cardinium* single-infected *S. furcifera* line; CW, *Cardinium* and *Wolbachia* double-infected *S. furcifera* line.

To further examine the effects of single and double infections on the bacterial microbiota, we examined the correlation among bacterial groups in the three lines across samples. At the phylum level, correlations in the three lines differed (Fig. 2A to C). In the uninfected U individuals, Acidobacteria was positively correlated with Patescibacteria and Verrucomicrobia and negatively correlated with Proteobacteria; Patescibacteria was positively correlated with Verrucomicrobia (Fig. 2A). In contrast, in the single-infected C individuals, Patescibacteria was negatively correlated with Verrucomicrobia; Bacteroidetes (including *Cardinium*) was negatively correlated with Actinobacteria and Patescibacteria and positively correlated with Verrucomicrobia (Fig. 2B). In the double-infected CW individuals, only three significant correlations were found, among which Bacteroidetes (including *Cardinium*) was negatively correlated with Proteobacteria (including *Wolbachia*) (Fig. 2C). The negative correlation between *Cardinium* and *Wolbachia* was confirmed by measuring the bacterial correlations in CW (Fig. 2D and E). *Cardinium* was positively correlated with *Halomonas*, *Curvibacter* and some other groups, and negatively correlated with others, including *Wolbachia* and *Pantoea* (Fig. 2D). *Wolbachia* was positively correlated with *Panacagrimonas*, *Marmoricola* and other groups, and negatively correlated with others, including *Cardinium* and *Aliihoeflea* (Fig. 2E).

### Combined *Cardinium* + *Wolbachia* infection reduces host fecundity over the single *Cardinium* infection.

The fecundity of the three lines was determined to compare the biological effects of *Cardinium* and the combined *Cardinium* + *Wolbachia* infection on the host. There was a significant difference in fecundity between the three lines (Kruskal-Wallis test: *P* < 0.001) (Fig. 2F). Compared with U, the fecundity of C was significantly reduced (Kruskal-Wallis test followed by Dunn's pairwise comparisons: *P* < 0.01), and the fecundity of CW was further reduced (*P* < 0.001). The double infection therefore reduced host fecundity more than the single infection.

### Relative densities of total bacteria, endosymbionts, and Acinetobacter in the three lines.

**FIG 3:**
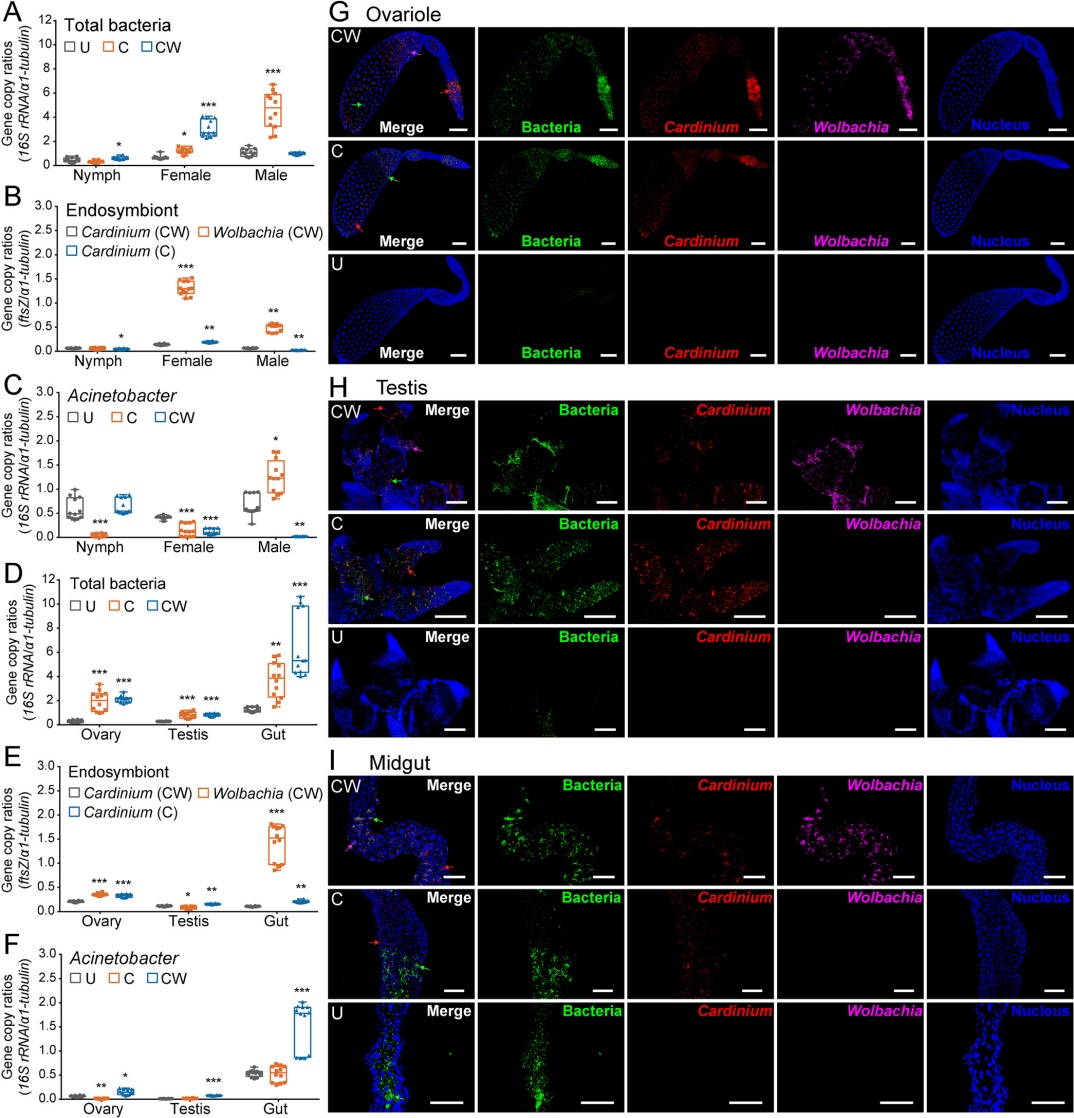
Densities and distributions of bacteria and endosymbionts in three *S. furcifera* lines. (A–F) Box plots representing the relative densities of total bacteria, endosymbionts (*Cardinium* and *Wolbachia*) and Acinetobacter across different developmental stages (A–C) and in adult tissues (D–F) of the three lines. Asterisks indicate significant difference between the infected group (C or CW) and uninfected group (U), *n *= 12; *, *P* < 0.05; **, *P* < 0.01; ***, *P* < 0.001. (G–I) Distributions of *Cardinium*, *Wolbachia* and nuclear DNA in the different adult tissues of the three lines. Ovariole (G), testis (H) and midgut (I). Bacteria, *Cardinium* and *Wolbachia* were stained as green, red and fuchsia, respectively, using specific probes. Corresponding arrows indicate the staining regions. Scale bars are 100 μm. U, uninfected *S. furcifera* line; C, *Cardinium* single-infected *S. furcifera* line; CW, *Cardinium* and *Wolbachia* double-infected *S. furcifera* line.

The densities of *Cardinium*, *Wolbachia* and the overall bacterial microbiota relative to the single copy gene *α1-tubulin* of *S. furcifera* were examined in whole insect body across different developmental stages (Fig. 3A to C), where the relative density refers to the density of total bacteria per host cell relative to this gene. For each line, the relative density of total bacteria was higher in adults than in nymphs. The density of all bacteria in nymphs also differed among the three lines (one-way ANOVA: *P* < 0.001) (Fig. 3A). Similar differences were found in females (*P* < 0.001) and in males (*P* < 0.001). The highest bacterial density was found in nymphs and females of the CW line and in males of the C line. The relative density of endosymbionts also varied with development stages (Fig. 3B). In both single- and double-infected lines (C and CW), the relative density of *Cardinium* was higher in females than in nymphs or males. The single-infected C line had a higher *Cardinium* density than the double-infected CW line for females (Kruskal-Wallis test followed by Dunn's pairwise comparisons: *P* < 0.01), while the opposite was found in nymphs (*P* < 0.05) and males (*P* < 0.01). For the double-infected CW, the relative densities of *Cardinium* and *Wolbachia* also differed in females (*P* < 0.001) and males (*P* < 0.01), but not in nymphs (*P* = 0.985). Furthermore, the relative density of Acinetobacter varied with host developmental stage and endosymbiont infection status (Fig. 3C).

The densities of total bacteria, endosymbionts, and Acinetobacter relative to *α1-tubulin* of *S. furcifera* varied in different adult tissues (Fig. 3D to F). For each line, the relative density of total bacteria was highest in the gut, lowest in the testis and intermediate in the ovary (Kruskal-Wallis test: U, *P* < 0.001; C, *P* < 0.001; CW, *P* < 0.001) (Fig. 3D). For each tissue, the presence of *Cardinium* or *Wolbachia* infections increased the bacterial density per host cell (Kruskal-Wallis test: ovary, *P* < 0.001; testis, *P* < 0.001; gut, *P* < 0.001) (Fig. 3E). In addition, the single-infected C always had a higher *Cardinium* density than the double-infected CW in the ovary (one-way ANOVA followed by Tukey's pairwise comparisons: *P* < 0.001), testis (Kruskal-Wallis test followed by Dunn's pairwise comparisons: *P* < 0.01) and gut (*P* < 0.01). For the double-infected CW, the relative density of *Wolbachia* was higher than that of *Cardinium* in the ovary (*P* < 0.001) and gut (*P* < 0.01), but the opposite was found in the testis (*P* < 0.01). The relative density of Acinetobacter was also different among tissues, and its density was highest in the gut for each line (Fig. 3F). In summary, endosymbiont density varied between nymphs and adults and also varied across different tissues, and the relative densities of total bacteria and Acinetobacter were affected by *Cardinium* and combined *Cardinium* + *Wolbachia* infections.

### Distributions of representative bacteria, *Cardinium*, and *Wolbachia* in the three lines.

Some bacteria that can be labeled with the universal probe were considered “representative bacteria,” and they were used as positive controls. The distribution of representative bacteria differed among the three *S. furcifera* lines and also differed in tissues of the same line. In the ovariole and testis, these bacteria were widespread in C and CW, but rare in U (Fig. 3G and H); in the midgut, they were widely distributed in all three lines (Fig. 3I). The distribution of *Cardinium* in single- and double-infected lines (C and CW) was similar. In the ovary, *Cardinium* was widely distributed but concentrated in the vitellarium of the ovariole (Fig. 3G); in the testis, *Cardinium* was more widely distributed in C than in CW (Fig. 3H); in the midgut, *Cardinium* was also distributed widely (Fig. 3I). Moreover, the distributions of *Cardinium* and *Wolbachia* varied in the double-infected CW. In the ovariole and testis, *Wolbachia* was more concentrated than *Cardinium* (Fig. 3G and H); in the midgut, the distribution of the two was similar, but *Wolbachia* was also more concentrated than *Cardinium* (Fig. 3I). These results showed differences in the distributions of representative bacteria and *Cardinium* between the lines and differences in the distributions of *Cardinium* and *Wolbachia* in CW and also highlighted tissue-specific distributions in some instances.

### Differential metabolites and KEGG enrichments.

**FIG 4:**
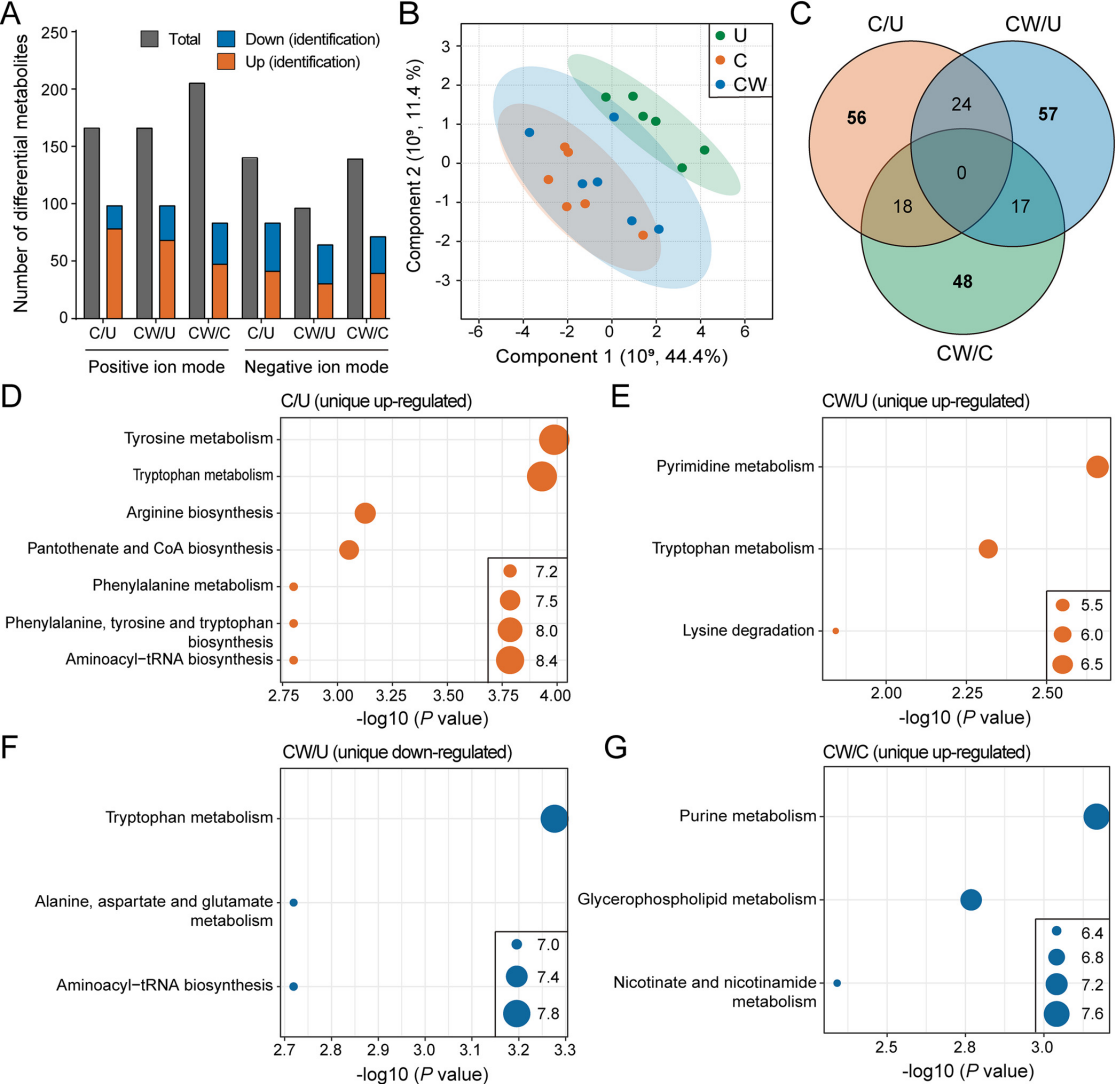
Metabolite analysis for the three *S. furcifera* lines. (A) The number of total differential metabolites and identified upregulated and downregulated differential metabolites for each comparison in positive ion mode and negative ion mode. (B) Principal Component Analysis (PCA) showing the sample clusters for the three lines. Coordinate axis represents the percent contribution of each principal component to the total variance. (C) Venn diagrams depicting the number of differentially expressed genes (DEGs) in the three comparisons. (D–G) Bubble diagrams indicating the Kyoto Encyclopedia of Genes and Genomes (KEGG) enrichment of differential metabolites. The differential metabolites include upregulated differential metabolites in the C/U comparison (D), upregulated (E) and downregulated (F) differential metabolites in the CW/U comparison and downregulated differential metabolites in the CW/C comparison (G). U, uninfected *S. furcifera* line; C, *Cardinium* single-infected *S. furcifera* line; CW, *Cardinium* and *Wolbachia* double-infected *S. furcifera* line.

There were more upregulated metabolites than downregulated metabolites in the three comparisons of C/U, CW/U and CW/C (Fig. 4A). The endosymbiont-infected lines (C and CW) and uninfected line (U) were significantly separated, while the samples of C and CW overlapped (Fig. 4B), which may be related to their common *Cardinium* infection.

Venn diagram analysis showed common and unique differential metabolites in the three comparisons (C/U, CW/U and CW/C) to highlight the effects of *Cardinium* and *Wolbachia* on the host's metabolism (Fig. 4C). The unique up- and downregulated metabolites in the three comparisons were enriched in some pathways as established through the KEGG database (Fig. 4D to G). KEGG enrichment analysis found that the upregulated metabolites in the C/U comparison were mainly related to amino acid metabolism (such as arginine biosynthesis), metabolism of cofactors and vitamins (pantothenate and CoA biosynthesis) and translation (aminoacyl-tRNA biosynthesis), while the downregulated metabolites were not significantly enriched (Fig. 4D). In the CW/U comparison, the upregulated metabolites were mainly related to nucleotide metabolism (pyrimidine metabolism) and amino acid metabolism (such as tryptophan metabolism) (Fig. 4E); the downregulated metabolites were mainly related to amino acid metabolism (such as alanine, aspartate and glutamate metabolism) and translation (aminoacyl-tRNA biosynthesis) (Fig. 4F). In the CW/C comparison, only the downregulated metabolites were enriched and mainly related to nucleotide metabolism (purine metabolism), lipid metabolism (glycerophospholipid metabolism) and metabolism of cofactors and vitamins (nicotinate and nicotinamide metabolism) (Fig. 4G). These results showed the effects of *Cardinium* and the combined *Cardinium* + *Wolbachia* infection on the host's metabolic levels, with *Cardinium* by itself perhaps promoting the host's metabolic functions while the combined infection had both promoting and suppressing effects.

### Association analysis reveals potential bacteria and metabolites related to fecundity differences in different host lines.

**FIG 5:**
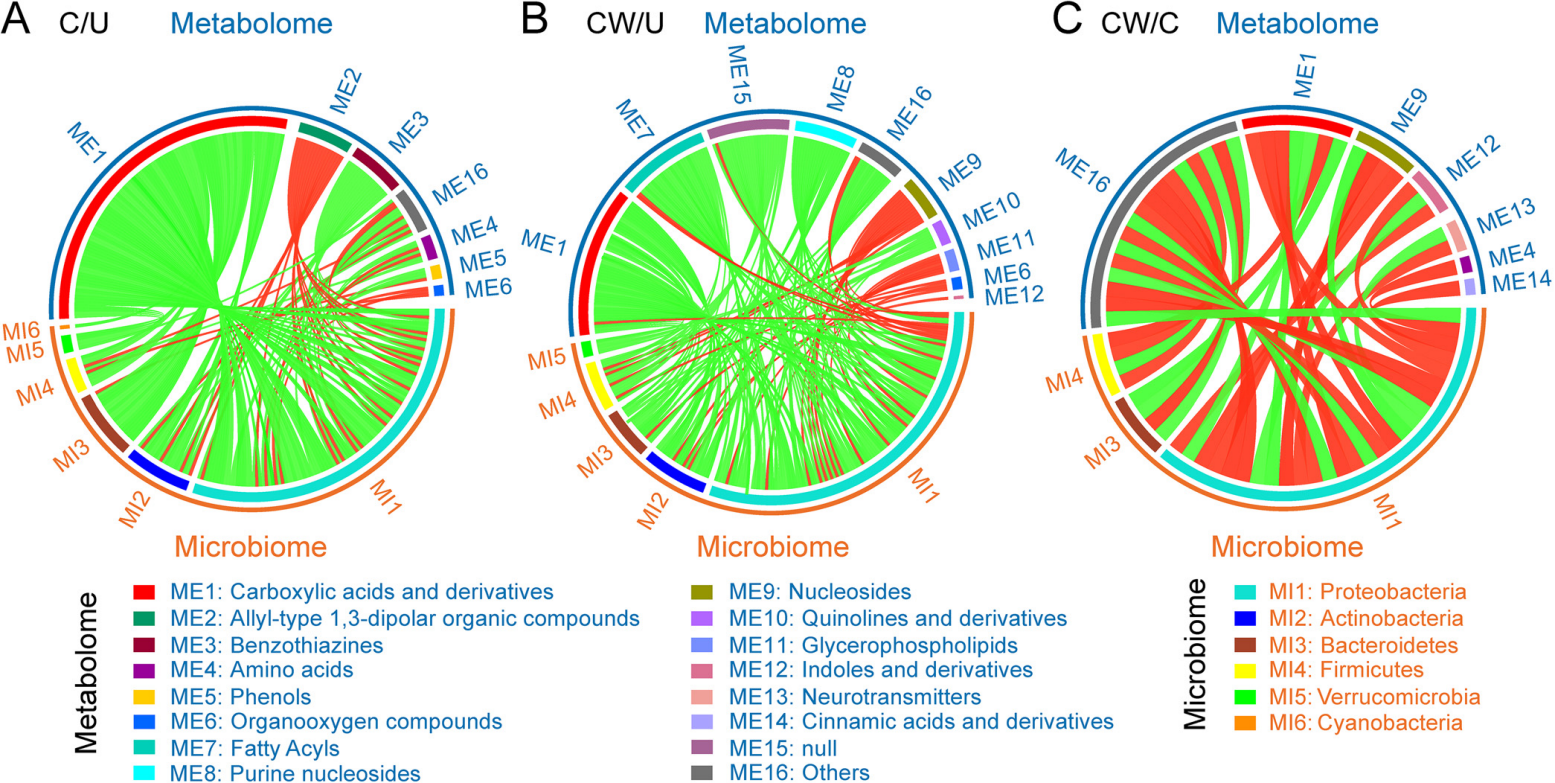
Correlation analysis of differential microbes and metabolites in the three group comparisons. Pairwise differential microbe-metabolite correlation analysis based on Spearman correlation analysis in C/U (A), CW/U (B) and CW/C (C) comparisons. Microbiome and metabolome data are shown only when coefficient *R*-value was > 0.9 or < −0.9 and *P* value was < 0.05. Red and green lines indicate positive and negative correlations, respectively. U, uninfected *S. furcifera* line; C, *Cardinium* single-infected *S. furcifera* line; CW, *Cardinium* and *Wolbachia* double-infected *S. furcifera* line.

Association analysis between microbiome and metabolome could help to understand the connections between bacterial physiological functions and biological phenotypes (Fig. 5A to C and Data set S1). In the C/U comparison, a total of 6 bacterial phyla (103 genera) were highly correlated with 7 metabolite classes (13 metabolites) (Fig. 5A). Among them, carboxylic acids and derivatives and benzothiazines were negatively correlated with these bacterial phyla; allyl-type 1,3-dipolar organic compounds and organooxygen compounds were positively correlated with the bacterial phyla; amino acids and phenols were positively or negatively correlated with the bacterial phyla. In the CW/U comparison, a total of 5 bacterial phyla (94 genera) were highly correlated with 10 metabolite classes (19 metabolites) (Fig. 5B). Among them, purine nucleosides and quinolines and derivatives showed a negative correlation with these bacterial phyla; organooxygen compounds, nucleosides, glycerophospholipids, and indoles and derivatives showed a positive correlation with the bacterial phyla, while carboxylic acids and derivatives, fatty acyls, and so on showed a positive correlation or negative correlation with the bacterial phyla.

10.1128/msystems.01516-21.5 DATA SET S1 Pairwise differential microbe-metabolite correlation based on Spearman correlation analysis for C/U, CW/U and CW/C comparisons in *S. furcifera*. U, uninfected *S. furcifera* line; C, *Cardinium* single-infected *S. furcifera* line; CW, *Cardinium* and *Wolbachia* double-infected *S. furcifera* line. Download Data Set S1, XLSX file, 0.03 MB. Copyright © 2022 Li et al. 2022 Li et al. https://creativecommons.org/licenses/by/4.0/ This content is distributed under the terms of the Creative Commons Attribution 4.0 International license.

In the above two comparisons, most bacteria were downregulated in both C/U and CW/U comparisons except for a few bacteria such as *Cardinium* in the C/U comparison, as well as *Cardinium* and *Wolbachia* in the CW/U comparison (Fig. 5A and B). In contrast, most metabolites were upregulated in both C/U and CW/U comparisons. These included gly-lys, l-phenylalanine and thioproperazine in the C/U comparison, and (+/-)-methoprene, arecoline and dihydrofarnesol in the CW/U comparison. Exceptions were 11-nitro-1-undecene and 9,12-octadecadienal in the C/U comparison and 2-linoleoyl-sn-glycero-3-phosphoethanolamine and deoxyadenosine in the CW/U comparison. In addition, a total of 3 bacterial phyla (14 genera) and 7 metabolite classes (13 metabolites) were highly correlated in the CW/C comparison (Fig. 5C). The numbers of upregulated and downregulated bacteria, as well as the numbers of upregulated and downregulated metabolites, were almost the same in the CW/C comparison. An exception was that *Cardinium* was downregulated and *Wolbachia* was upregulated in the CW/C comparison. These results showed a high correlation between the bacteria and metabolites and showed that the bacterial changes induced by endosymbionts.

## DISCUSSION

The present study showed that both single *Cardinium* and double *Cardinium* + *Wolbachia* infections reduced bacterial diversity and changed bacterial community structure in both developmental stages (nymph, female, and male of whole insect body) and adult tissues (ovary, testis, and gut). Endosymbiont density varied with host development and specific tissue and endosymbionts affected the densities of total bacteria and Acinetobacter. The distributions of these bacteria in adult tissues were also visualized. Both single and double infections changed the correlation pattern between bacteria, and *Cardinium* negatively correlated with *Wolbachia* in the double infected line. In double-infected *T. truncatus* (27) and D. melanogaster (28), a similar correlation exists between *Cardinium* and *Wolbachia*. The double infection reduces host fecundity more than the single infection. In *N. lugens*, *Cardinium* also reduces fecundity, whereas *Wolbachia* promotes fecundity (14, 17). Lines also differed in levels of some metabolites related to host fecundity. *Cardinium* mainly upregulated metabolic pathways while the combined infection had both up- and downregulated effects. Moreover, association analysis found that many bacteria had strong correlations with differential metabolites and indicated that the bacterial changes induced by endosymbionts may lead to changes in many functions which could be related to host fecundity. These results were obtained from a comparison of three lines that had been developed from a common genetic background and backcrossed to further homogenize the genetic background, although given the challenges of creating the lines it was not possible to do similar line comparisons across different host genetic backgrounds.

### Effects of *Cardinium* and *Wolbachia* on bacterial microbiota in *S. furcifera*.

Our results showed that both the single and double infection can reduce bacterial diversity and change bacterial community structure (including bacterial corrections). The bacterial diversity of C and CW was similar in most developmental stages and tissues (except for males), which indicates that both *Cardinium* and the combined *Cardinium* + *Wolbachia* infections reduce bacterial diversity, and *Cardinium* may have a dominant influence on metabolic pathways in the double-infected line (CW). Endosymbionts have also been reported to reduce bacterial diversity in other pests. Artificially introduced *Cardinium* and *Wolbachia* reduced the diversity of resident bacteria in *N. lugens* (32) and Aedes aegypti (33), respectively; some natural *Wolbachia* strains decreased bacterial diversity in D. melanogaster (34), *L*. *striatellus* (35), and ticks (36). However, this reduction effect does not always occur, such as in Anopheles coluzzii (37). In pests infected with two endosymbionts, one of them usually has a dominant effect on the bacterial microbiota (38–40). For example, in drosophilid flies, which were infected with both *Wolbachia* and *Spiroplasma*, *Wolbachia* had a much greater impact on microbes (38). Overall, our results showed that the bacterial diversity of single- and double-infected lines (C and CW) was similar in most developmental stages and tissues, thus single and double infections have similar effects on bacterial diversity in *S. furcifera*.

There are likely to be several classes of mechanisms by which endosymbionts affect bacterial microbiota. First, the endosymbionts and other bacteria depend on limited nutrition and space to survive and may be in a competitive relationship (41, 42), which would reduce the abundance of less competitive bacteria. Endosymbionts are involved in an indirect interaction with other bacteria given that they live inside the cell, unlike gut bacteria (34, 43, 44). *Cardinium* contains a Por secretion system and a putative phage-derived protein secretion system (45, 46), and may secrete compounds into a host’s extracellular environment to regulate bacterial abundance. *Wolbachia* contains a type 4 secretion system, which may secrete specific compounds impacting host biology and regulating the interaction between *Wolbachia* and other bacteria (47–49). Second, endosymbionts can induce host immune responses (such as antimicrobial peptides) which in turn may regulate bacterial diversity (50–52). Transinfected *Wolbachia* strains can upregulate the host's immune response (53, 54). Although natural *Wolbachia* strains often have no obvious effects on immune responses (55, 56), *Wolbachia* infection can significantly upregulate reactive oxygen species (ROS) levels of pest species (57). In *S. furcifera*, the immune response in the uninfected line (U) was downregulated compared with the infected lines (C and CW), and perhaps *Cardinium* or *Wolbachia* may generate an intestine-specific immune response (such as ROS) to destabilize bacterial diversity (58).

### Variation in bacterial microbiota in different developmental stages and different tissues of *S. furcifera*.

Bacterial microbiota in *S. furcifera* differed in nymphs and adults as well as in adult tissues (Fig. 1 and S3). Bacterial microbiota dynamically change with developmental stages in many insects (9, 13, 32, 59). These changes may be related to changes in the host's physiological environment during metamorphosis to which the bacterial microbiota need to adapt (9). The differences in the distribution of bacteria across tissues of each line are consistent with findings on other planthoppers, including *N. lugens* and *L. striatellus* where the distribution of commensal bacteria vary across the intestine, ovary, testis, head, and fat body (32, 35). The highest relative density of bacteria in the gut probably reflects the strong connection between this tissue and the external environment. Tissue-specific structures and characteristics (such as pH) would also determine which bacteria can invade particular tissues, while bacterial dispersal ability may play an important role in a host's microbial ecology (58).

### Effects of *Cardinium* and the combined *Cardinium* + *Wolbachia* infections on the metabolic levels in *S. furcifera*.

A Venn diagram analysis revealed the KEGG enrichment pathways of unique differential metabolites in the three comparisons (C/U, CW/U and CW/C). Only upregulated pathways were observed in the C/U comparison and only downregulated pathways were observed in the CW/C comparison, which suggests that the single infection upregulates metabolic activity further than the double infection, perhaps due to *Wolbachia* and *Cardinium* having opposing effects on metabolism. The upregulated metabolic pathways in the *Cardinium*-infected line may reflect the nutritional requirements of this bacterium when it is the only endosymbiont present in a host. In contrast, *Cardinium* and *Wolbachia* induced similar changes in metabolic capacity in *A. albopictus* (60), A. aegypti (61), the spider *Hylyphantes graminicola* (62), and D. melanogaster (63), so the opposing effects of the two endosymbionts in *S. furcifera* runs counter to this pattern.

Some of the changes in metabolic pathways in the present study probably contributed to fecundity differences among the different lines. For example, arginine biosynthesis was upregulated in the C/U comparison, and an increase in arginine may have led to a decrease in the fecundity of the single-infected line (C). Arginine participates in various metabolic processes such as the ornithine cycle in many species, and it is a precursor of several substances, including proline, glusate glutamate, urea, and nitric oxide (64–66). However, high levels of arginine can decrease fecundity in D. melanogaster (67). Nicotinate and nicotinamide metabolism, which differed in the CW/C comparison, may also influence fecundity. Nicotinamide participates in the formation of coenzymes and is closely related to many metabolic processes such as glycolysis (68, 69). In aphids, changes in nicotinamide affect the host's reproduction and survival (70). Association analysis showed strong correlations between specific differential metabolites and bacterial changes in the different comparisons (Fig. 5), suggesting a potential link between bacteria, metabolites and fecundity affected by endosymbionts.

### Possible connections and next steps.

**FIG 6:**
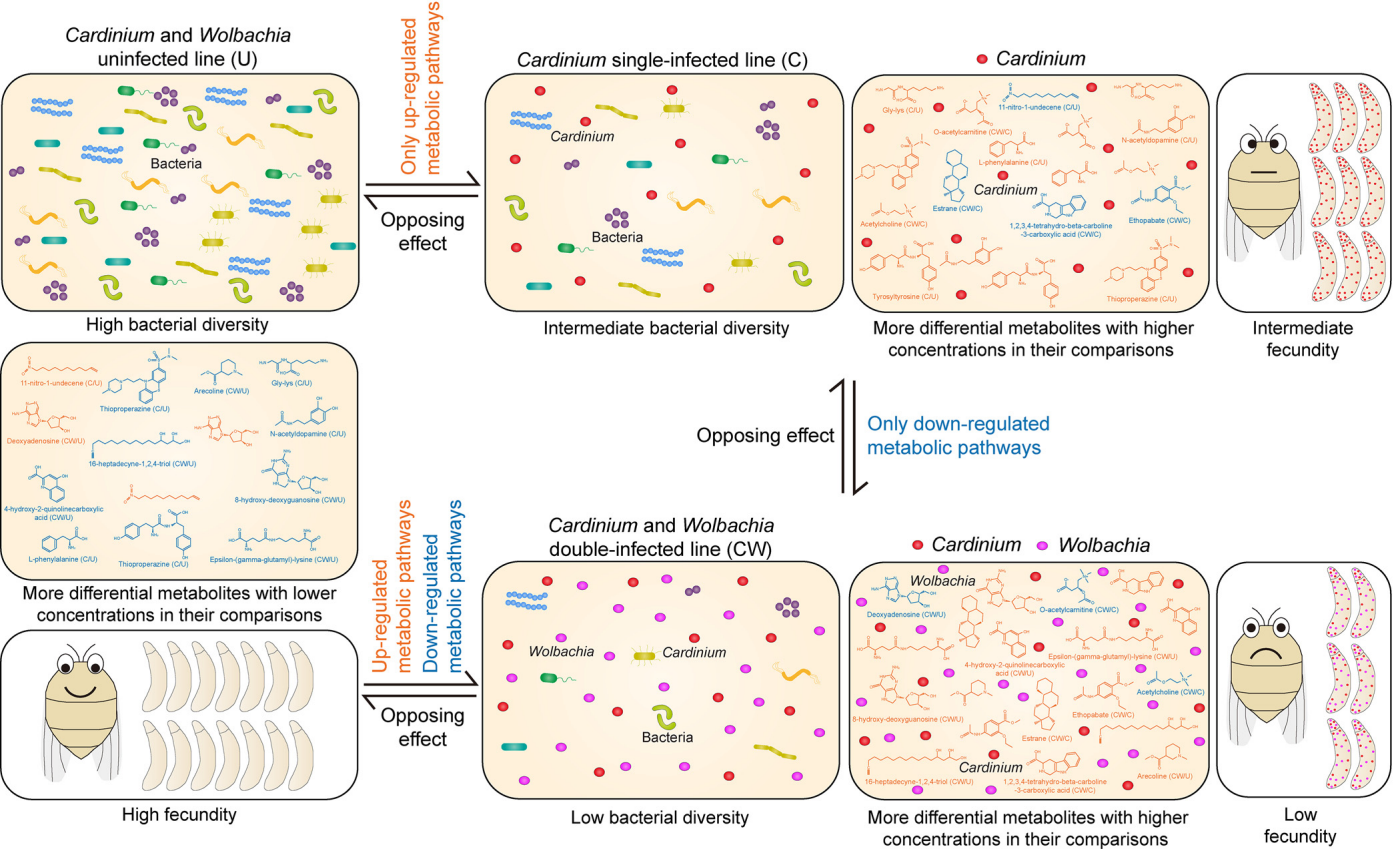
Model for the potential correlations between bacterial diversity, metabolic response and host fecundity under the infection of endosymbionts. The differences in endosymbionts, bacterial diversity, metabolites, host fecundity and their potential correlations were described. The number of bacteria represents the level of bacterial diversity. The representative differential compounds in the three lines are described. Orange and blue, respectively, indicate the compounds with relatively high and low concentrations in their comparisons between the two lines. The compound names were marked below the structural formula, and the names of the two lines being compared were marked behind the name. The fecundity effects of the three lines were expressed by the number of eggs. Orange and blue texts next to the arrows, respectively, indicate the upregulated and downregulated KEGG metabolic pathways at the arrow directions.

The potential connections between the bacteria, metabolites, and host fecundities under endosymbiont infections (Fig. 6) provide an opportunity for further experiments. We hypothesize that individuals in the uninfected line (U) may not need to provide extra nutrition to endosymbionts, resulting in a high bacterial diversity because less competitive bacteria can persist, and allowing the host to have a high fecundity. In the single-infected line (C), bacterial density decreases as does host fecundity due to the nutritional requirements of *Cardinium* which also results in higher levels of differential metabolites. Individuals in the double-infected line (CW) may need adequate nutrition for the survival of both endosymbionts which further decreases bacterial diversity. In addition, metabolic pathways may be affected by interactions between *Cardinium* and *Wolbachia*, further decreasing fecundity. Therefore, host-supplied metabolites and nutrients for endosymbionts are critical. Many of the effects of these metabolically limited endosymbionts may be exerted through manipulation of the microbiome; despite being unable to synthesize many compounds themselves, endosymbionts may trigger changes in the synthesis of compounds by bacteria in other tissues. These ideas could be tested by changing the nutritional environment wherein the planthoppers reside, such as through various conditions in which plant hosts grow (71). They could also be further tested by applying antibiotics that specifically affect some bacterial groups and then measuring changes in relative density to see if there is evidence of competitive interactions (72). Finally, it may be possible to apply specific inhibitors of metabolic pathways to see if this alters the relative abundance and diversity of the bacterial groups.

In summary, we found that *Cardinium* and the combined *Cardinium* + *Wolbachia* infections reduced bacterial diversity as well as changing bacterial community structure and affecting metabolism which may connect to negative fitness effects of the endosymbionts on their host. Given that endosymbionts have both positive and negative effects on their hosts, it will be interesting to undertake these types of comparisons on other host-endosymbiont systems where both positive and negative effects of the endosymbionts on host fitness have been demonstrated. It would also be worthwhile testing for the differences described here in other genetic backgrounds of the planthopper host.

## MATERIALS AND METHODS

### White-backed planthopper lines and endosymbionts infection status.

In this study, a total of three *S. furcifera* lines with different endosymbiont infection status were developed, namely, a *Cardinium* and *Wolbachia* double-infected line (CW), a *Cardinium* single-infected line (C), and an uninfected line (U). A field *S. furcifera* population was collected from Yunnan Province, China in 2017. All nine collected female adults were co-infected with *Cardinium* and *Wolbachia* by PCR (i.e., suggesting a 100% infection rate consistent with previous research [29]). One adult female of the field population was used to establish an isofemale line double-infected by *Cardinium* and *Wolbachia* (CW). CW was fed on rice seedlings treated with 1g/L tetracycline for at least three consecutive generations. From the second generation of tetracycline treatment onwards, we randomly selected female adults to rear on fresh rice seedlings individually and tested their infection status after they had laid eggs. We then tested the offspring of any female that appeared single-infected by *Cardinium*. This process led to a single-infected *Cardinium* isofemale line being established from the third generation of treatment. Similarly, an isofemale line (U) uninfected by *Cardinium* and *Wolbachia* was selected and obtained by feeding CW on rice seedlings treated with 0.5g/L rifampicin for three consecutive generations. A *Wolbachia* single-infected *S. furcifera* line (W) could not be obtained because *Wolbachia* was always lost prior to *Cardinium* when lines were treated with antibiotics. To eliminate antibiotic effects, all *S. furcifera* lines were reared on normal rice seedlings at 26°C and 16/8h (light/dark) cycle for at least 18 months, which also ensured that the bacterial microbiota could be stabilized in the host. Furthermore, to minimize nuclear differences between the lines, C females or CW females were backcrossed to U males for three consecutive generations prior to the experiments being started.

### Fecundity determination.

The fecundity of the three *S. furcifera* lines was tested according to a previous method (14). A female and male that emerged on the same day were mated in a cuvette with fresh rice seedlings. After 5 days, they were transferred to another fresh rice seedling and allowed to lay eggs for 7 days, during which time the rice seedlings were replaced once to ensure sufficient space for spawning. Forty pairs were set up for each line, and replicates where the female died prematurely during spawning were discarded. Following a previous method (73), fecundity was determined by dissecting rice seedlings and counting the number of eggs buried in leaf sheaths. Host fecundity was analyzed nonparametrically by using Kruskal–Wallis tests with *post hoc* Dunn's pairwise comparisons. IBM SPSS Statistics (Version 24.0, IBM Corporation, Armonk, NY, USA) was used, and *P* values of <0.05 were considered significant.

### *16S rRNA* gene amplicon sequencing and data assembly.

To determine bacterial microbiota in the three *S. furcifera* lines, the samples of whole insect body at different developmental stages were collected for *16S rRNA* gene amplicon sequencing. Sample collections followed a previous method (32). The developmental stages considered were 3rd instar nymphs and adults at their 5th day of development (females and males). In addition, *16S rRNA* sequencing was carried out on dissected adult tissues, involving the ovary and gut of 5th day females and the testis of 5th day males. For sample collections, 10 individuals or tissues were collected as a pool and three pools (biological replicates) were obtained per line. To remove potential bacteria located on the surface of individuals or tissues, samples were first washed with 75% ethanol for 90 s and then rewashed three times with sterile deionized water.

Genomic DNA was extracted using Qiagen DNeasy blood and tissue kit (Qiagen, Germany). The V3-V4 hypervariable regions of *16S rRNA* gene were amplified by specific primers (forward: 5′-CCTAYGGGRBGCASCAG-3′, reverse: 5′-GGACTACNNGGGTATCTAAT-3′) (32). The DNA amplicons were purified and estimated using 2% agarose gel electrophoresis, and then quantified by QuantiFluor™-ST (Promega, Madison, WI, USA) fluorometry. All purified DNA products were prepared as a paired-end (2 × 250 bp) DNA library and sequenced on the Illumina HiSeq 2500 platform (Biozeron, Shanghai, China).

The methods for data assembly were similar to those used in previous research (32). Raw FASTQ files were acequired to obtain valid sequences based on the barcode sequence information, and QIIME (version 2, https://qiime2.org) and in-house Perl programs were used for demultiplexing and optimization. Operational taxonomic unit (OTU) clustering was performed on nonrepeating sequences with a 97% similarity cutoff by USEARCH (version 10, http://drive5.com/uparse/), and chimeric sequences were removed to obtain representative OTU sequences by UCHIME (version 7.1, http://drive5.com/uparse/). All optimized sequences were mapped to representative sequences to generate an OTU table according to the 97% similarity.

### Microbial analysis.

In order to improve the accuracy of sequenced data, low-quality and uninformative OTU data were filtered out by MicrobiomeAnalyst (https://www.microbiomeanalyst.ca/). Optimized OTU data met the criteria that at least 10% of the samples of an OTU contained at least 2 reads.

Alpha diversity was computed to characterize changes of bacterial species richness (i.e., number of bacterial species) and evenness (i.e., relative bacterial abundance) among different *S. furcifera* lines. A total of five alpha diversity indexes were computed: Chao1 and ACE indexes were used to reflect species richness, while Fisher, Shannon and Simpson indexes were used to reflect bacterial evenness (MicrobiomeAnalyst, https://www.microbiomeanalyst.ca/). We have focused on two diversity indexes (Chao1 and Fisher) in the results, as the other diversity indexes provided similar patterns. The distribution of alpha diversity estimates was tested for normality by Shapiro-Wilk tests. Data that met this requirement were analyzed parametrically by using one-way analysis of variances (ANOVA) with *post hoc* Tukey's pairwise comparisons. Data that were not normally distributed were analyzed nonparametrically by using Kruskal–Wallis tests with *post hoc* Dunn's pairwise comparisons. Parametric and nonparametric analyses were performed with IBM SPSS Statistics (Version 24.0, IBM Corporation, Armonk, NY, USA), and *P*-values <0.05 were considered significant.

Beta diversity was compared by permutational multivariate analysis of variance (PERMANOVA) and principal coordinate analyses (PCoA) of different developmental stages and adult tissues (MicrobiomeAnalyst, https://www.microbiomeanalyst.ca/). Differences among communities were expressed as Bray-Curtis dissimilarity distances. The relative abundance of bacteria at phylum and genus levels were plotted to compare differences in bacterial structure. In addition, core microbiome analysis was performed by detecting the occurrence frequency (i.e., prevalence) of bacteria in the body samples of each *S. furcifera* line.

To further determine the impacts of endosymbionts on the host microbiome, we tested the co-occurrence of bacteria at the phylum level within each line by computing Kendall's correlation coefficients among individual samples. The correlation of *Cardinium* and *Wolbachia* was also examined within the CW line.

### Density and distribution of microbes.

The relative densities of total bacteria, endosymbionts (*Cardinium* and *Wolbachia*) and Acinetobacter in different developmental stages and adult tissues were characterized. The same samples as used for *16S rRNA* gene sequencing were assessed for the densities of total bacteria using quantitative PCR (qPCR). Three biological replicates (pools) were obtained for each life stage (i.e., each developmental stage of whole insect body or each adult tissue), and 4 technical replicates were performed for each biological replicate. The specific primers (forward: 5′-AGRGTTYGATYMTGGCTCAG-3′; reverse: 5′-TGCTGCCTCCCGTAGGAGT-3′) of bacteria *16S rRNA* gene (74), specific primers (forward: 5′-AGCATGTGCAAGCCCAAGAAGG-3′; reverse: 5′-TGCTTTTGGCGGCAGTGGTT-3′) of *Cardinium ftsZ* gene (30), specific primers (forward: 5′-TTATCACAGCAGGGATGGGT-3′; reverse: 5′-TTTTTTCTTTTGCTCCTTTATCTTTAACTA-3′) of *Wolbachia ftsZ* gene (30) and specific primers (forward: 5′-CGTGCGTAGGCGGCTTCTTA-3′; reverse: 5′-TTCGTACCTCAGCGTCAGTATTAGG-3′) of Acinetobacter *16S rRNA* gene (32) were used to determine gene copy numbers. As these were single-copy genes, copy numbers of the bacteria based on them were normalized against the single-copy *α1-tubulin* gene (forward: 5′-CAACAACTACGCCAGAGG-3′; reverse: 5′-CCGAATGAGTGGAAGATGAG-3′) (75) of *S. furcifera* to estimate relative densities of the two endosymbionts (i.e., bacterial density per host cell). The above qPCR experiments were performed according to the conditions in the primer references and the instructions of ChamQ Universal SYBR qPCR Master Mix (Vazyme Biotech, Nanjing, Jiangsu, China). The relative densities of bacteria and endosymbionts were compared using parametric or nonparametric analyses depending on whether data met normality requirements (as for the alpha diversity measures). *P*-values <0.05 were considered significant.

The distributions of *Cardinium*, *Wolbachia* and representative bacteria in the three *S. furcifera* lines were visualized by fluorescence *in situ* hybridization (FISH) following our previous methods (14). Briefly, the tissues of adults on the5 to 7th day after emergence were carefully dissected, labeled with specific probes with fluorescent labels, stained with DAPI (4′,6-diamidino-2-phenylindole), mounted with anti-fluorescence quenching mounting medium, and then scanned with a Leica TCS SP8 laser confocal microscope. Two Cy5 5′-end-labeled specific probes (C162: 5′-ATCTTTCCAGCATGCGCT-3′; C587: 5′-CAATCGCAGTTCTAGCGTTA-3′) of *Cardinium 16S rRNA* gene (14) and two rhodamine 5′-end-labeled specific probes (W1: 5′-AATCCGGCCGARCCGACCC-3′; W2: 5′-CTTCTGTGAGTACCGTCATTATC-3′) of *Wolbachia 16S rRNA* gene (76) were used. One FITC 5′-end-labeled probe (Bac: 5′-GCTGCCTCCCGTTAGGAGT-3′) of the *16S rRNA* gene some bacteria (including *Cardinium*, *Wolbachia* Enterobacter, *Serratia*, *Brachybacterium*, and *Pseudonocardia*) was used as a positive control, and these bacteria that can be labeled were named “representative bacteria.”

### Metabolomics determination.

Nontargeted metabolomics was performed by Liquid Chromatography-Mass Spectrometry (LC-MS) to determine metabolite differences between the three *S. furcifera* lines. Thirty whole female bodies were collected as one pool on the 5th day after emergence and six biological replicates (pools) for each line were tested. The samples were frozen in liquid nitrogen for 15 min and metabolites were extracted. Then metabolite profiles were detected by high performance liquid chromatography (Waters 2D UPLC, Waters, USA) and high-resolution mass spectrometer (Q exactive HF, Thermo Fisher Scientific, USA). Metabolite data for positive ion mode and negative ion mode were collected to improve the coverage and accuracy of metabolites. Meanwhile, the same volume of samples from six biological replicates were mixed into quality control (QC) samples, and nine needles of blank solvent were interspersed to monitor instrument status and evaluate data quality during the metabolite determination.

### Metabolite analysis.

Quality control and compound identification followed previous methods (77). Compounds with low quality were deleted by setting a threshold for the variation coefficient of relative peak area greater than 30% in all QC samples. The number of ions with a variation coefficient ≤ 30% for ionic strength in all QC samples covered more than 90% of the metabolites, indicating that the fluctuations were mostly small. Metabolites were identified by using Compound Discoverer (version 3.0, Thermo Fisher Scientific, USA) combined with BGI library, mzCloud and ChemSpider (HMDB, KEGG, LipidMaps). More metabolites were identified in positive ion mode than in negative ion mode, so subsequent analyses employed the metabolite data in the positive mode.

To screen and compare the metabolites among different *S. furcifera* lines, VIP-values in the first two principal components of Partial Least Squares Discriminant Analysis (PLS-DA) model ≥ 1, fold changes ≥ 1.2 or ≤ 0.83 and Benjamini-Hochberg adjusted *P*-values <0.05 were applied. The differences between the three lines were determined by PLS-DA. Venn diagrams based on the above parameters were plotted to depict the differential metabolites in the three comparisons (C/U, CW/U and CW/C). The differential metabolites from the above comparisons were subjected to the Kyoto Encyclopedia of Genes and Genomes (KEGG) (https://www.kegg.jp/) functional enrichment analysis to understand functional differences.

### Association analyses of differential bacteria and differential metabolites.

Pairwise correlations between differential bacteria and differential metabolites in each comparison (C/U, CW/U and CW/C) were computed by using M^2^IA (http://m2ia.met-bioinformatics.cn/). Since the microbiome and metabolome assessments had different biological replicate numbers, two replicates of the microbiome were used to each replicate of the microbiome. First, correlation coefficients were calculated between bacteria and metabolites using Spearman pairwise correlation analyses within each line. Correlations of interest were those with coefficient values greater than 0.9 or less than −0.9 and *P*-values less than 0.05. Then the differential bacteria and differential metabolites between the three comparisons (C/U, CW/U and CW/C) were subjected to univariate analyses. The identification of differential metabolites was the same as above, while differential bacteria were identified by comparing their relative abundance in groups being compared with Student's *t*-tests, and *P*-values <0.05 were considered significant. The patterns linking differential bacteria and differential metabolites were visualized by circus plots (Data set S1).

### Sequencing and metabolite output profile.

A total of 1,582,852 counts were obtained from *16S rRNA* gene sequences of 54 samples, with an average of 29,312 counts for each sample (Table S1). Among them, the counts for developmental stages and adult tissues were 780,491 and 783,984, respectively. A high level of Good's coverage (≥99.4%) indicated a high accuracy of the sequencing results. A total of 485 OTUs were obtained after quality control at 97% species sequence similarity, with OTUs belonging to 10 phyla, 18 classes, 55 orders, 89 families and 186 genera.

10.1128/msystems.01516-21.4 TABLE S1 Sequencing and alpha diversity index profile of microbiome for all samples in the three *S. furcifera* lines. Download Table S1, DOCX file, 0.02 MB. Copyright © 2022 Li et al. 2022 Li et al. https://creativecommons.org/licenses/by/4.0/ This content is distributed under the terms of the Creative Commons Attribution 4.0 International license.

After quality control, a total of 2314 and 753 compounds were detected in positive ion mode and negative ion mode, respectively, of which 1139 and 408 compounds were identified, respectively (Data set S2). Results from the positive ion mod e with more identified metabolites was used for subsequent analysis.

10.1128/msystems.01516-21.6 DATA SET S2 Metabolite profile for all samples in three *S. furcifera* lines. POS, positive ion mode; NEG, negative ion mode. U, uninfected *S. furcifera* line; C, *Cardinium* single-infected *S. furcifera* line; CW, *Cardinium* and *Wolbachia* double-infected *S. furcifera* line. Download Data Set S2, XLSX file, 1.6 MB. Copyright © 2022 Li et al. 2022 Li et al. https://creativecommons.org/licenses/by/4.0/ This content is distributed under the terms of the Creative Commons Attribution 4.0 International license.

### Data availability.

The data from *16S rRNA* sequencing have been deposited in the National Center for Biotechnology Information (NCBI) Sequence Read Archive (SRA) under the BioProject accession number PRJNA737327. The profiles of all metabolites are shown in Data set S2.
