# Supplementary figures and images for: Endosymbionts Reduce Microbiome Diversity and Modify Host Metabolism and Fecundity in the Planthopper Sogatella furcifera
Source: mSystems. 2022 Mar 30;7(2):e01516-21. doi: 10.1128/msystems.01516-21 (PMC9040572; doi:10.1128/msystems.01516-21)

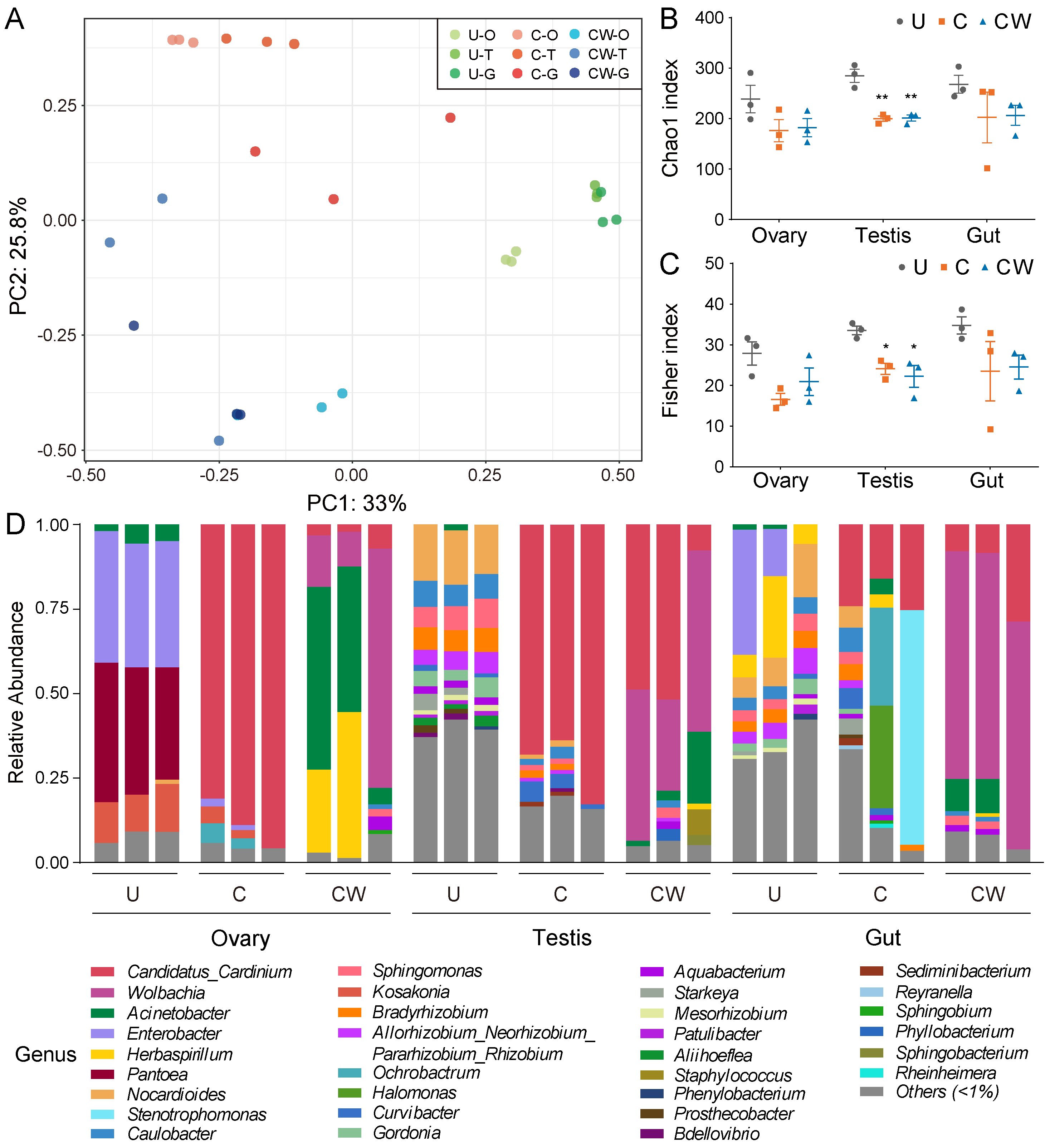

Supplement: FIG S3 [file msystems.01516-21-sf003.tif]

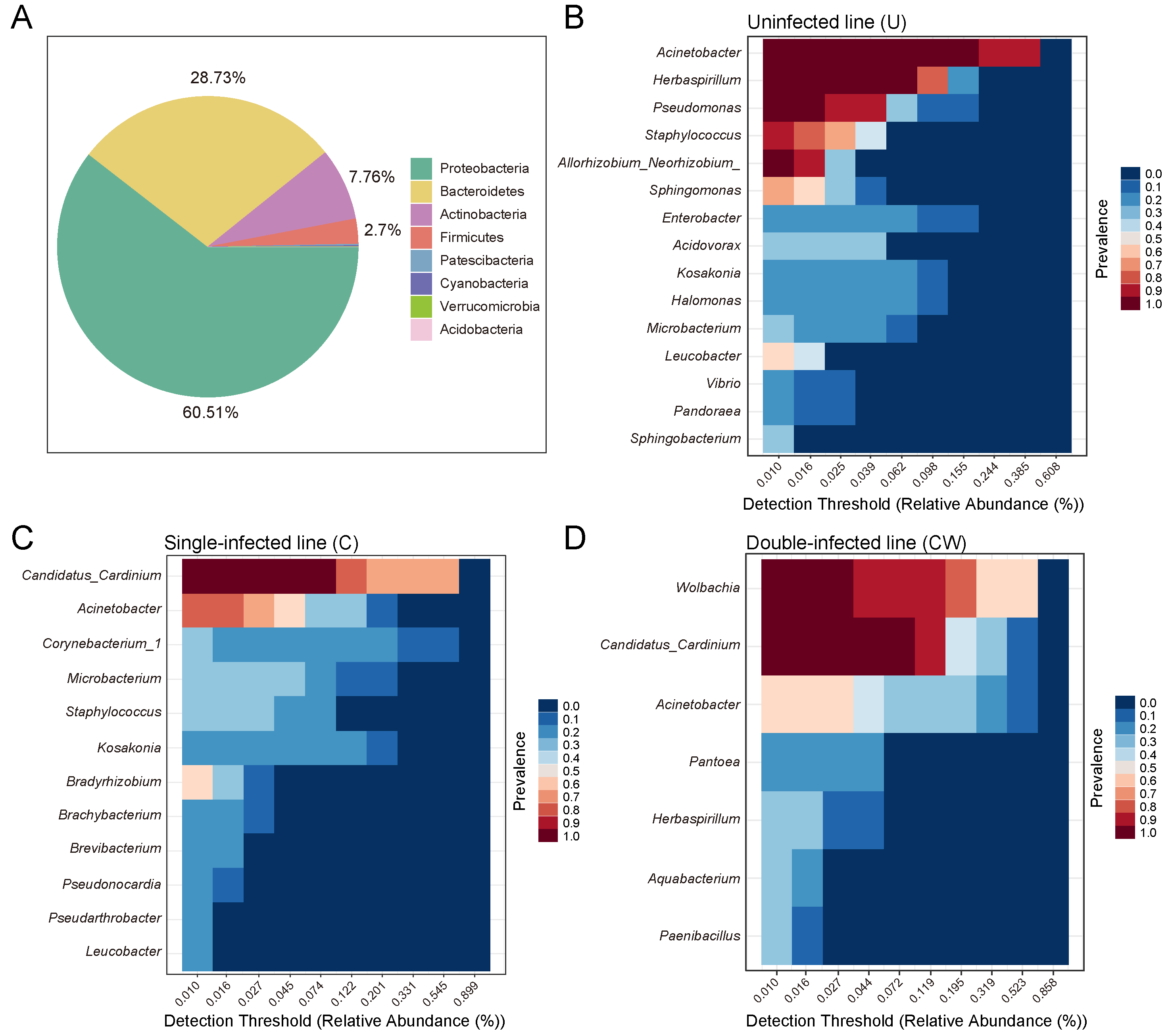

Supplement: FIG S1 [file msystems.01516-21-sf001.tif]

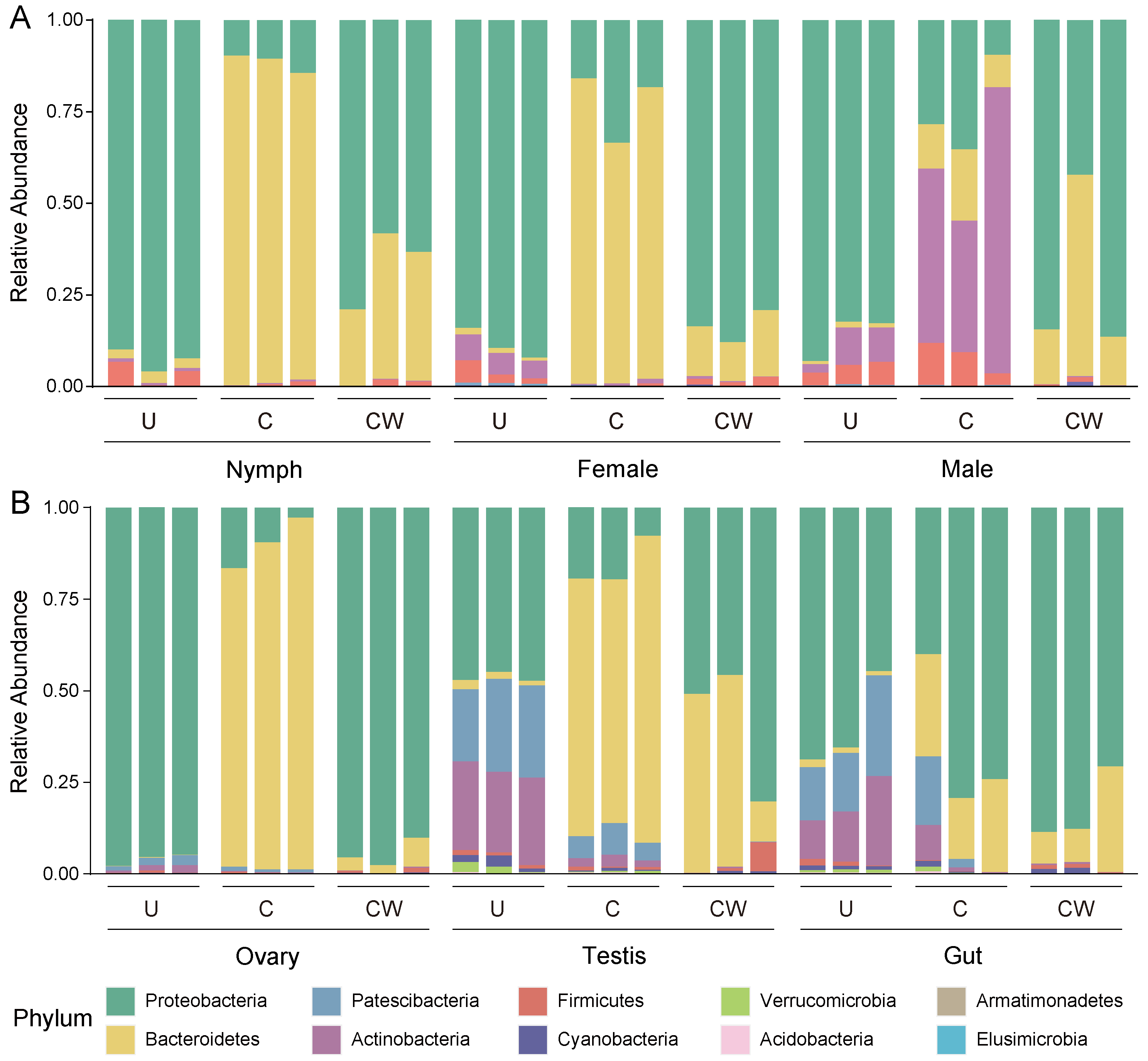

Supplement: FIG S2 [file msystems.01516-21-sf002.tif]
