# Supplementary material for: Endosymbionts Reduce Microbiome Diversity and Modify Host Metabolism and Fecundity in the Planthopper Sogatella furcifera
Source: mSystems. 2022 Mar 30;7(2):e01516-21. doi: 10.1128/msystems.01516-21 (PMC9040572; doi:10.1128/msystems.01516-21)
Supplement: TABLE S1 [file msystems.01516-21-st001.docx]

**TABLE S1** Sequencing and alpha diversity index profile of microbiome for all samples in the three *S. furcifera* lines*^a^*.

| Sample types | Read counts | Good's coverage | Observed OTUs | Chao1 index | Shannon index | Simpson index | ACE index | Fisher index |
| --- | --- | --- | --- | --- | --- | --- | --- | --- |
| U-N | 29296±15.77 | 99.43±0.12 | 298.33±15.28 | 317.36±14.33 | 3.38±0.16 | 0.92±0.01 | 314±14.96 | 46.91±3.09 |
| U-F | 29310.33±13.27 | 99.66±0.04 | 344.33±10.87 | 362.57±6.28 | 3.57±0.14 | 0.93±0 | 365.16±7.05 | 54.95±2.04 |
| U-M | 29311±13.44 | 99.6±0.02 | 345.33±13.27 | 378.86±8.4 | 3.22±0.39 | 0.88±0.04 | 370.04±10.74 | 55.12±2.51 |
| C-N | 29306.67±24.57 | 99.8±0.01 | 171.33±15.97 | 207.72±7.73 | 0.82±0.18 | 0.24±0.05 | 207.6±8.24 | 24.16±2.63 |
| C-F | 29326.67±1.25 | 99.78±0.02 | 132.33±30.18 | 200.06±17.6 | 0.92±0.19 | 0.38±0.09 | 197.78±20.24 | 17.97±4.78 |
| C-M | 29327.33±0.94 | 99.74±0.03 | 219.67±20.07 | 259.32±12.91 | 2.48±0.6 | 0.77±0.14 | 257.99±9.2 | 32.32±3.47 |
| CW-N | 29325.33±3.77 | 99.78±0.03 | 153.33±43.99 | 204.64±37.28 | 1.77±0.57 | 0.66±0.15 | 204.69±38.63 | 21.41±6.91 |
| CW-F | 29326.67±1.89 | 99.77±0.02 | 137±4.55 | 193.89±20.01 | 1.13±0.55 | 0.45±0.21 | 190.3±16.14 | 18.62±0.72 |
| CW-M | 29325.67±3.3 | 99.85±0 | 118±19.82 | 158.18±17.51 | 1.13±0.47 | 0.47±0.14 | 156.38±19.32 | 15.71±3.06 |
| U-O | 29325±2.16 | 99.75±0.04 | 192.67±24.23 | 238.74±38.39 | 2.19±0.11 | 0.81±0.02 | 247.34±39.98 | 27.86±4.04 |
| U-T | 29226.67±99.8 | 99.78±0.03 | 226.67±9.03 | 284.96±18.57 | 3.34±0.1 | 0.91±0.01 | 263.11±19.36 | 33.52±1.53 |
| U-G | 29308±6.38 | 99.8±0.05 | 233.67±16.94 | 268.13±25.29 | 3.16±0.08 | 0.89±0.02 | 265.07±21.82 | 34.72±2.98 |
| C-O | 29324±4.32 | 99.81±0.03 | 123.67±12.92 | 176.2±30.86 | 0.64±0.26 | 0.21±0.1 | 172.25±22.49 | 16.58±2.02 |
| C-T | 29323±4.97 | 99.81±0.03 | 171±11.78 | 199.55±7.32 | 1.55±0.35 | 0.47±0.12 | 200.8±11.06 | 24.09±1.92 |
| C-G | 29322.33±4.19 | 99.8±0.06 | 164.67±64.98 | 202.36±71.25 | 2.08±0.9 | 0.71±0.19 | 202.14±69.96 | 23.48±10.27 |
| CW-O | 29321±8.52 | 99.8±0.03 | 151±29.68 | 181.87±25.79 | 1.75±0.17 | 0.67±0.13 | 189.14±26.78 | 20.92±4.79 |
| CW-T | 29304±17.91 | 99.81±0.01 | 159.33±23.7 | 201.04±8.5 | 1.61±0.36 | 0.63±0.05 | 189.11±18.88 | 22.25±3.8 |
| CW-G | 29307.67±6.94 | 99.79±0.02 | 173.33±25.77 | 206.27±28.38 | 1.43±0.38 | 0.51±0.04 | 212.47±21.09 | 24.53±4.18 |

*^a^*Data were shown as the mean ± SEM. U, uninfected *S. furcifera* line; C, *Cardinium* single-infected *S. furcifera* line; CW, *Cardinium* and *Wolbachia* double-infected *S. furcifera* line; N, nymph; F, female; M, male; O, ovary; T, testis; G, gut.
